# The effect of graft application and simvastatin treatment on tibial bone defect in rats. A histological and immunohistochemical study^1^

**DOI:** 10.1590/s0102-865020190040000008

**Published:** 2019-05-06

**Authors:** Nihat Laçin, Bozan Serhat İzol, Ebru Gökalp Özkorkmaz, Buşra Deveci, Mehmet Cudi Tuncer

**Affiliations:** I Assistant Professor, Department of Oral and Maxillofacial Surgery, Faculty of Dentistry, University of Katip Çelebi, İzmir, Turkey. Technical procedures, manuscript preparation and writing, final approval.; II PhD, Research Assistant, Department of Periodontology, Faculty of Dentistry, University of Bingöl, Turkey. Technical procedures, manuscript preparation and writing, final approval.; III Assistant Professor, Department of Histology and Embryology, Faculty of Medicine, University of Dicle, Diyarbakır, Turkey. Technical procedures, histological examinations, manuscript preparation and writing, final approval.; IV PhD, Research Assistant, Department of Periodontology, Faculty of Dentistry, University of Dicle, Diyarbakir, Turkey. Technical procedures, manuscript preparation and writing, final approval.; V PhD, Professor, Department of Anatomy, Faculty of Medicine, Dicle University, Diyarbakır, Turkey. Technical procedures, histological examinations, manuscript preparation and writing, final approval.

**Keywords:** Osteogenesis, Simvastatin, Osteopontin, Osteonectin, Rats

## Abstract

**Purpose:**

To evaluate histologically and immunohistochemically the bone regeneration after application of simvastatin on tibial bone defects in rats.

**Methods:**

Sixty Wistar albino rats were divided into 3 groups as control (6 mm tibial bone defect), defect + graft (allograft treatment), and defect + graft + simvastatin (10 mg/kg/day) for 28 days.

**Results:**

Histopathological examination revealed inflammation in control group (defect group), congestion in blood vessels, and an increase in osteoclast cells. In defect + graft group, osteoclastic activity was observed and osteocyte cells were continued to develop. In defect + graft + simvastatin group, osteocytes and matrix formation were increased in the new bone trabeculae. Osteopontin and osteonectin expression were positive in the osteclast cells in the control group. Osteoblasts and some osteocytes showed a positive reaction of osteopontin and osteopontin. In defect + graft + simvastatin group, osteonectin and osteopontin expression were positive in osteoblast and osteocyte cells, and a positive expression in osteon formation was also seen in new bone trabeculae.

**Conclusion:**

The simvastatin application was thought to increase bone turnover by increasing the osteoinductive effect with graft and significantly affect the formation of new bone.

## Introduction

 Bone defects constitute a difficult problem. Different grafting materials have been used to eliminate osseous defects. The aim of using these materials are osteoconductive and osteoinductive. Bone defect, which occurs due to various reasons (such as trauma, infection or other factors) leads to serious problems^1^. For instance, cortical grafts provide a durable and rigid structure but they have no ability to increase osteogenesis^2^ and as they lack of vascularisation, necrotic bone cannot repair itself. The primary advantage of cancellous bone and bone marrow is that they are able to significantly enhance osteogenesis. These abilities depend on the fact that they have viable cells that can transform into osteoblasts as well as those that induce osteogenesis^3^. The autograft is used as a bone graft due to its osteoinductive and osteoconductive properties. Allograft was also used as a bone substitution because it prevented donor site morbidity^4^.

 Many studies have indicated that statins act towards remodeling and formation of bone tissue and are thus osteoinductive substances. Mundy *et al.* ^5^ reported from the studies on rodents that statins have anabolic effects on bone by the stimulation of tissue formation. Osteoblasts and marrow adipocytes are originated from a common mesenchymal progenitor and adipogenic agents are known to suppress osteoblast differentiation^6^.

 Simvastatin is a modification of lovastatin serving as a rate-limiting enzyme in cholesterol synthesis^7^. Simvastatin is not well absorbed, and less than 5% of an oral dose reaches the systemic circulation. Concentrations of statins in bone marrow have not been well establishedbut osteoblasts and osteoclasts may be exposed to very low concentrations of statins when taken orally^8^. Simvastatin enhances alkaline phosphatase activity and mineralization, as well as increases the expression of bone sialoprotein, osteocalcin and type I collagen, and it was shown to have anti-inflammatory effect by decreasing the production of interleukin-6 and interleukin-8^9^. Simvastatin was reported to enhance osteoblastic activity and inhibit osteoclastic activity so, increase bone formation rate, volume and strength^10^.

 Osteopontin (OPN) secreted by the osteoblasts, is one of the major non-collagenous proteins^11^. Studies concerning the expression of mRNA show a low expression of OPN during the maturation of the rat calvarium and the tibia in early bone formation. The osteopontin secretion increases and reaches its highest level in 14-day-old bone. OPN mRNA decreases and remains low but detectable in bone cells along the periosteal and endosteal surfaces and in the spongiosa of the tibia in young adult bone^12^. Bone defect healing is the process of bone formation. It has been reported that spontaneous recovery of the bone defect and bone regeneration ability occurs but takes time under appropriate physiological environmental conditions. New bone formation, the reduction of blood flow in the region of the fracture band to strengthen and harden the new bone is reported to occur slowly due to lack of calcium and phosphorus^12^ ^-^ ^15^. Healing process of bone involves the chemotaxis of mesenchymal cells, their proliferation and differentiation into osteoblastic cells and the production and mineralization of extra- cellular matrix (ECM) by osteoblasts. It has been found that these critical cellular events are tightly regulated by appropriate signal molecules, such as growth factors and cytokines.

 The aim of this study was to investigate application of graft with simvastatin treatment on created tibial bone defects of rats using histopathological and immunohistochemical methods. 

## Methods

 This study was approved by the Ethics Committee for Animal Experimentation of the Faculty of Medicine at Dicle University,Turkey. 

 Sixty male Wistar albino rats weighting 260-290 g were used in this study. Wistar Albino rats were maintained under 22±1°C and 12 h light/dark cycles with *ad libitum* access to standard pelleted food and water. All rats at the end of experiment were healthy and no difference in food/water consumption and body weight gain between experimental and control rats were observed. Every single surgical methodology and the consequent care and healing of the animals utilized as a part of this investigation were in strict understanding with the National Institutes of Health (NIH Publications No. 85-23, revised 1985) rules for animal care. 

### Tibial bone defect model

#### Sedation and surgical procedure

 The animals were anesthetized by intraperitoneal injection 5 mg/kg of Ketamine (Ketalar^®^, Eczacıbası, Turkey) and xylazine HCL (Rompun^®^, Bayer, Turkey). The skin was shaved and scrubbed with an antiseptic solution (1% iodine). After exposing the right proximal tibia of each animal, a standardized 6. 0 mm diameter non-critical bone defect was created by using a motorized drill under irrigation with saline solution^8^ ^-^ ^10^. The material used in our study was Biograft^®^ HT (IFGL Bio Ceramics) which contains 40% β-Tri Calcium Phosphate with 60% porous biphasic synthetic Hydroxyapatite. This material is an alloplast with granule size of 350-500 µm with osteoconductive properties. So, alloplastic material (Biograft-HT^®^) was placed in defect area in group 2 and group 3. 

 Three groups (20 rats per group) were arranged as below:

1. Control group (Defect group): Tibial bone defect was created on the first day of study and rats were kept immobile for 28 days .
2. Defect + graft group: Six mm tibial bone defect with allograft treatment was applied on the first day of study and rats were kept immobile for 28 days .
3. Defect + graft + simvastatin group: Alloplastic bone graft was placed in the tibial bone defect on the first day of study and simvastatin treatment was applied. All rats were kept immobile for 28 days. Simvastatin (Wako Pure Chemical Industries, Osaka, Japan) was diluted by physiological saline solution and administrated orally using a gastric tube. The dose of simvastatin was 10 mg/kg per day starting from the first day of study to the end.

At the end of the study, animals were sacrified by decapitation. The skin, as well as all of the soft tissues surrounding the tibia bone were removed. The samples were fixed with 10% neutral buffered formalin solution and decalcified with 5% EDTA (Ethylene diamin tetra acetic acid). After rinsing with tap water, the samples were dehydrated in increasing concentrations of ethanol and embedded in paraffin. Tissue sections of 4-6 µm thickness were prepared in the transverse plane and stained using Hematoxylin-eosin for light microscopy examination.

### Immunohistochemical staining

 Antigen retrieval was done in microwave (Bosch^®^, 700 watt) for 3min x90^o^C. They were subjected to a heating process in a microwave oven at 700 watts in a citrate buffer (pH 6) solution for proteolysis. Sections were washed in 3x5 min PBS and incubated with hydrogen peroxide [K-40677109 ,64271 Hydrogen peroxide (H_2_O_2_) Dortmudt+Germany, MERCK] (3ml %30 Hydrogen peroxide (H_2_O_2_) + 27ml methanol) for 20 min. Sections were washed in 3x5 min PBS min and blocked with Ultra V Block (lot: PHL150128, Thermo Fischer, Fremont, CA, USA) for 8 min. After draining, primary antibodies were directly applied to sections distinctly Osteonectin (SPARC), Catalog #:33-5500, 1:100, Thermo Fischer, Fremont, CA, USA, Osteopontin monoclonal antibody 1:100, (MA5-17180), Thermo Fischer, Fremont, CA, USA. Sections were incubated and left overnight at 4^o^C. Sections were washed in 3x5 min PBS and then incubated with Biotinylated Secondary Antibody (lot: PHL150128, Thermo Fischer, Fremont, CA, USA) for 14 min. After washing with PBS, Streptavidin Peroxidase (lot: PHL150128, Thermo Fischer, Fremont, CA, USA) was applied to sections for 15 min. Sections were washed in 3x5 min PBS and DAB (lot: HD36221, Thermo Fischer, Fremont, CA, USA) were applied to sections up to 10 min. Slides showing reaction was stopped in PBS. Counter staining was done with Harris’s Haematoxylin for 45 sec, dehydrated through ascending alcohol and cleared in xylene. Product Number: HHS32 *SIGMA,* Hematoxylin Solution, Harris Modified, Sigma-Aldrich, 3050 Spruce Street, Saint Louis, MO 63103, USA. Slides were mounted with Entellan^®^ (lot: 107961, Sigma-Aldrich, St. Louis, MO, United States) and examined under light microscope (Zeiss, Germany).

 All morphological changes in osteoblast, osteocyte and osteoclast cells, inflammation, congestion in blood vessels, new bone formation were detaily noted. The intensity of these changes were graded from 0 to 4 (Fig. 1).

**Figure 1:**
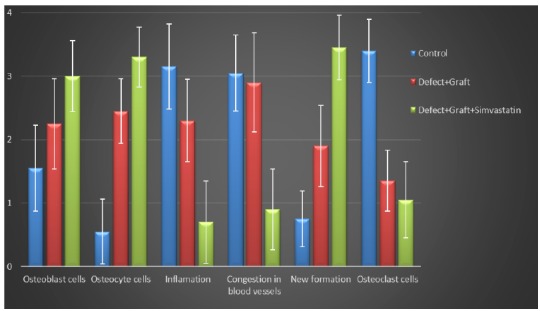
Histogram showing statistical analysis of histological parameters in all groups. The quantification of all parameters: **0:** no change, **1:** too week, **2:** week, **3:** middle, **4:** strong. (Scoring was determined by examining histological parameters in 15 different regions within the microscope field).

### Statistical analysis

 Statistics and analyzes were performed using the SPSS 22.0 for Windows computer package program. In the analysis of the data, Kruskall-Wallis and Mann-Whitney U non-parametric statistical tests were used in the intergroup comparisons depending on the variables and the results were given as the mean ± standard deviation and mean rank. And, the results were considered statistically significant for p=0 with Kruskal-Wallis test and p < 0.05 with Mann-Whitney U test (Table 1).

**Table 1:** Comparison of all groups with Kruskal-Wallis and Mann-Whitney U test. Data are expressed as the mean ± standard deviation and mean rank (*p=0 with Kruskal-Wallis test, **p<0.05 with Mann-Whitney U test, * and ** statistically significant result).

Parameter	*Groups*	*n*	*Mean±SD*	*Mean Rank*	*Kruskal-Wallis Test value*	*Mann-Whitney U comparisons for groups (p<0,05)*
*Osteoblast cells*	*(1) Control (Defect)*	*20*	*1.55±0.68*	*17.08*	*27.270* *p=0	**(2) **(3)
	*(2) Defect+Graft*	*20*	*2.25±0.71*	*30.12*		**(1) **(3)
	*(3) Defect+Graft+Simvastatin*	*20*	*3.00±0.56*	*44.30*		**(1) **(2)
*Osteocyte cells*	*(1) Control (Defect)*	*20*	*0.55±0.51*	*10.50*	*49.069* *p=0	**(2) **(3)
	*(2) Defect+Graft*	*20*	*2.45±0.51*	*33.65*		**(1) **(3)
	*(3) Defect+Graft+Simvastatin*	*20*	*3.30±0.47*	*47.35*		**(1) **(2)
*Inflamation*	*(1) Control (Defect)*	*20*	*3.15±0.67*	*46.20*	*41.998* *p=0	**(2) **(3)
	*(2) Defect+Graft*	*20*	*2.30±0.65*	*33.45*		**(1) **(3)
	*(3) Defect+Graft+Simvastatin*	*20*	*0.70±0.65*	*11.85*		**(1) **(2)
*Congestion in blood vessels*	*(1) Control (Defect)*	*20*	*3.05±0.60*	*41.45*	*39.218* *p=0	**(3)
	*(2) Defect+Graft*	*20*	*2.90±0.78*	*38.80*		**(3)
	*(3) Defect+Graft+Simvastatin*	*20*	*0.90±0.64*	*11.25*		**(1) (2)
*New bone formation*	*(1) Control (Defect)*	*20*	*0.75±0.44*	*12.38*	*48.717* *p=0	**(2) (3)
	*(2) Defect+Graft*	*20*	*1.90±0.64*	*29.45*		**(1) (3)
	*(3) Defect+Graft+Simvastatin*	*20*	*3.45±0.51*	*49.68*		**(1) (2)
*Osteoclast cells*	*(1) Control (Defect)*	*20*	*3.40±0.50*	*50.50*	*44.499* *p=0	**(2) (3)
	*(2) Defect+Graft*	*20*	*1.35±0.48*	*22.98*		*(1)
	*(3) Defect+Graft+Simvastatin*	*20*	*1.05±0.60*	*18.02*		*(1)

## Results

### Histopathologic examinations

 In the histopathologic examinations findings were as follows for each group:

1. Control group (Defect group): Intensive infiltration, obstruction of blood vessels, increase in osteoclasts and necrotic changes were observed in the defect area. Degeneration of osteoblast cells and picnotic changes in osteocyte cell nuclei were also observed (Fig. 1a).
2. Defect + graft group: Reduction of inflammation, obstruction of arteries, increased osteblastic activity, osteoinductive effect, progression of osteocyte development and increased collagen fibers in connective tissue were our histopathological findings (Fig. 1b).
3. Defects + graft + simvastatin group: We found an increase in osteoinductive effect in osteoblasts, maturation of lacunar osteocyte cells, an increase in osteogenic matrix formation and a decrease in osteoclastic activity (Fig. 1c).

**Figure 1a:**
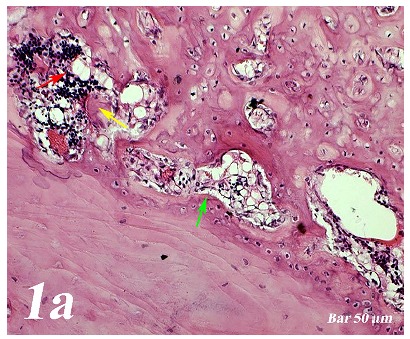
Haematoxylin-eosin staining (Control group). Intensive infiltration in the defect area, congestion in vessels (*yellow arrow*), increase in osteoclasts (*red arrow*) and necrotic changes, degeneration of osteoblast cells and picnotic changes in osteocyte cell nuclei (*green arrow*). Scale bar = 50 μm.

**Figure 1b:**
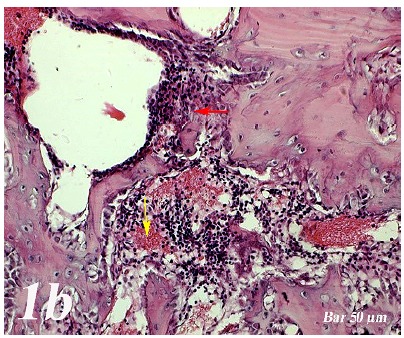
Haematoxylin-eosin staining (Defect + graft group). Decrease of inflammation in the defect area and congestion in the vessels (*yellow arrow*), an increase in osteoblastic activity, osteoinductive effect and osteocyte (*red arrow*) development. Increased connective tissue collagen fibers. Scale bar = 50 μm.

**Figure 1c:**
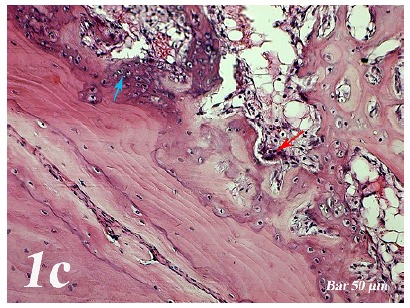
Haematoxylin-eosin staining (Defect + graft + simvastatin group). An increase in osteoinductive effect in osteoblasts (*blue arrow*), osteogenic matrix formation (*red arrow*) and osteogenic matrix with a decrease in osteoclastic activity. Scale bar = 50 μm.

### Immunohistochemical examinations

1. Control group (Defect group): Osteonectin expression was positive in osteoclast cells and some inflammatory cells in the defect area. Positive osteopontin reaction was observed in fibroblast and collagen fibers around congested blood vessels (Fig. 2a). Positive osteopontin expression was observed in osteoclast cells and inflammatory cells in the defect area while negative osteopontin expression was observed in osteoblast cells (Fig. 3a).
2. Defect + graft group: In the defect area, increase in connective tissue fibers and fibroblast cells were seen in histological sections. Osteonectin expression was positive in osteoclast and osteoblast cells (Fig. 2b). An increase in connective tissue fibers and cellular structures within the defect area were observed, while osteoclast cells were decreased. And, osteopontin expression was positively seen in sections. Positive osteopontin expression was observed in osteoblast cells with increased inductive effect in osteoblast cells at the periphery of the tibial bone trabecula (Fig. 3b).
3. Defect + graft + simvastatin group: New bone trabeculae in the graft area had begun to take shape. Osteonectin expression was positive in connective tissue fibers and fibroblast cells in this area. Both osteonectin expression was observed in osteoblast and osteocyte cells in new bone trabeculae. And, osteonectin expression was positive in osteon structures. Osteonectin expression levels and osteoclast cells decreased together. (Fig. 2c). In the graft area, positive osteopontin expression was seen in osteoblast and osteocyte cells of new bone trabeculae, osteopontin expression was significant in osteoclast cells and osteon structures (Fig. 3c).

**Figure 2a:**
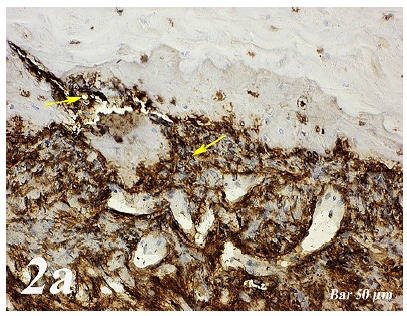
Osteonectin immunostaining (Control group). Positive expression of osteonectin in osteoclast cells and some inflammatory cells within the defect area (*yellow arrow*). Scale bar = 50 μm.

**Figure 2b:**
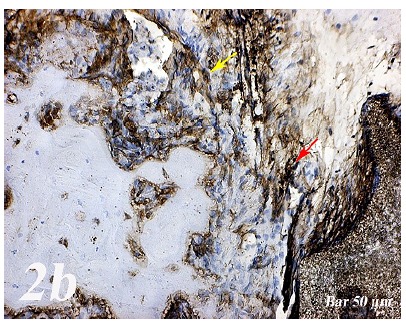
Osteonectin immunostaining (Defect + graft group). An increase in connective tissue fibers and fibroblast cells and positive osteonectin expression in the region of the graft in the defect area (*yellow arrow*), positive osteonectin expression in osteoclast cells and some osteoblast cells (*red arrow*). Scale bar = 50 μm.

**Figure 2c:**
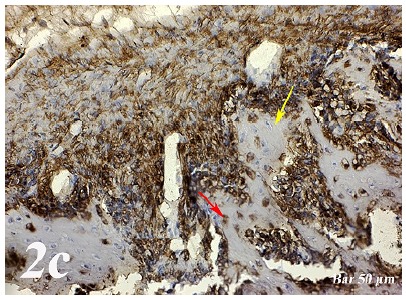
Osteonectin immunostaining (Defect + graft + simvastatin group). Positive osteonectin expression of connective tissue fibers and fibroblast cells due to fusion in the new bone trabecula together with the graft (*yelow arrow*). An increase in osteonectin expression in osteoblasts and osteocyte cells (*red arrow*) in new bone trabeculae, whereas osteonectin expression in osteon structures, A decrease in osteonectin expression levels with decreased osteoclast cells (*black arrow*). Scale bar = 50 μm.

**Figure 3a:**
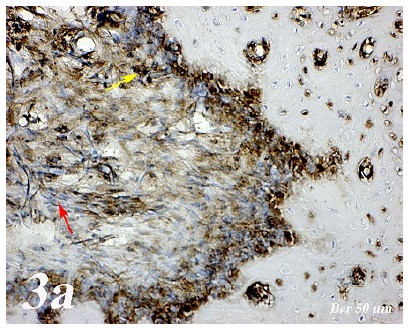
Osteopontin immunostaining (Control group). Osteopontin expression in osteoclast cells and inflammatory cells in the defect area (*yellow arrow*), negative osteopontin expression in osteoblast cells (*red arrow*). Scale bar = 50 μm.

**Figure 3b:**
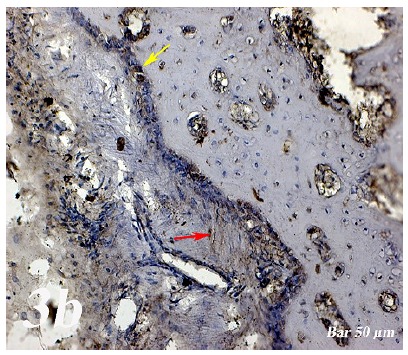
Osteopontin immunostaining (Defect + graft group). An increase in connective tissue fibers (*red arrow*) and cellular structures within the defect area, a decrease in osteoclast cells, positive osteopontin expression in osteoblast cells (*yellow arrow*). Scale bar = 50 μm.

**Figure 3c:**
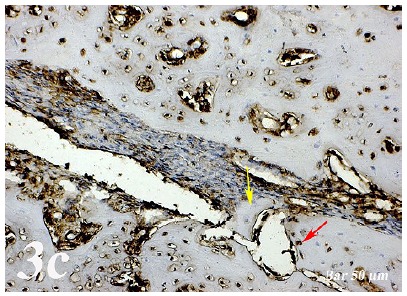
Osteopontin immunostaining (Defect + graft + simvastatin group). In the graft area, in new bone trabeculae (*yellow arrow*), positive osteopontin expression in osteoblast and osteocyte cells (*red arrow*). Scale bar = 50 μm.

### Statistical results

 The results of comparison of the parameters (osteoblast, osteocyte and osteoclast cells, inflammation, congestion in blood vessels, new bone formation) in all groups are shown in Table 1. When comparing the groups with these two statistical tests, significant results were obtained between the groups in all parameters. In parallel with the histological and immunohistochemical results of our study, it was also seen that simvastatin application increased new bone formation and decreased the level of inflammation among all groups (*p=0 with Kruskal-Wallis test , **p < 0.05 with Mann-Whitney U test) (Table 1). In addition, it was also seen that simvastatin application increased osteoblastic activity in all groups (*p=0 with Kruskal-Wallis test , **p < 0.05 with Mann-Whitney U test) (Table 1). 

## Discussion

 In segmental fractures, the longitudinal blood circulation of the fragments that are in between is compromised due to the loss of connections at proximal and distal points and the severe soft tissue trauma added to that adversely influences circulation, thereby increasing the risk of inadequate fracture healing^16^. Different sites are used to evaluate the bone healing of defects created in rats, such as tibia^17^, calvaria^18^ and jaws^19^. The tibial proximal epiphysis has a medial face that is suitable for inducing bone defects, as it has a wide and slightly convex surface that is devoid of muscle insertion. It has been shown by some researchers that experimental osseous defect repair caused different bone reactions due to biomaterial in rat tibial bone and used for the evaluation of bone healing. Bone grafts are often required to provide support, fill gaps and improve biological repair in skeletal damage^12^ ^,^ ^13^ ^,^ ^20^.

 Different bone graft materials have been used for bone regeneration, closure of osteotomy openings, and alveolar augmentation in oral and maxillofacial surgeons^21^ ^,^ ^22^. Natural coral-derived grafts and synthetic bone graft materials are used in alveolar crest elevation, intra-bone defects, material loss fractures, facial bone defects, orthognathic surgery, and maxillary sinus ground^22^ ^-^ ^24^.

 Simvastatin exerts anti-inflammatory effects, induces angiogenesis and promotes endothelial cell growth. It also regulates bone regeneration by promoting osteoblast function and inhibiting osteoclast cell function. A number of studies have demonstrated the potential for statins to increase bone regeneration^9^ ^,^ ^25^. Skoglund *et al*.^26^ reported that simvastatin induced the activation of the cells involved in the fracture repair process and thus increased bone callus size. In this study, osteoclastic activity was observed in the graft treated group and osteocyte cells were seem to be developed. In the simvastatin group, osteoplastic activity was increased and osteocytes and matrix formation with new bone trabeculae and inflammation and congestion in blood vessels were decreased.

 Osteonectin is secreted by osteoblast cells during bone formation. Osteopontin is expressed in bone and is thought to control the growth of mineral crystals^27^. Osteopontin (OPN) is a non-collagenous matrix protein. OPN is known to play a role in cell adhesion, migration, survival, and bone remodeling^28^ ^,^ ^29^. Research in animal models indicates that OPN deficiency alters the functionality of multiple cell types, resulting in delayed early vascularization, altered matrix organization, and late bone remodeling^30^. In our study, osteopontin and osteonectin expression were positive in some inflammatory cells in osteoclast cells in defect regionOsteoblasts and some osteocytes in the graft group showed a positive reaction of osteonectin and osteopontin (Figs. 2b and 3b). Osteonectin and osteopontin expression were positive expression in osteoblast osteocytes and bone trabeculae in simvastatin group (Figs. 2c and 3c).

 Clinical studies have shown that simvastatin administration reduces the risk of fractures and osteoporosis in patients^31^. Many investigators have used simvastatin by administering different doses. The regeneration of the calvarial bone, which was injected with simvastatin into the tissue at a dose of 1,5,10 mg/kg/day for 5 days, was studied^5^. Another investigator made subcutaneous injections of up to 50 mg/kg/day at higher doses to measure the possible effect of simvastatin on implant osseointegration in rabbits^32^. Kheirallah *et al*.^33^ reported that increased bone formation and resorption at high doses of simvastatin, while simvastatin at low dose reduces bone formation and increases bone resorption. Simvastatin loaded into calcium sulfate cement has shown to increase bone regeneration in a rat calvarial defect at eight weeks compared to non-simvastatin treated rats, despite the induction of a robust inflammatory response^34^. 

 In our study, rapid development of new bone trabeculae on the 28th day of the defect region was observed and also increase in osteoblastic and osteoclastic activities were accelerated by graft material and simvastatin application. Additionally, it provided support for the repair of bone tissue. In osteoblasts with fusion of graft in bone trabecula, matrix development and new bone trabeculae have emerged by increasing osteogenic effect in inductive and osteocyte cells. Positive osteopontin expression in osteoblast and osteocyte cells, decrease in osteoclast cells and prominence in osteon structures were observed in new bone trabeculae in graft area suggesting us that cell adhesion and bone remodeling are present.

 Despite the results, there are a few limitations in clinical contribution. Graft materials used to prevent damage to the bones caused by defect in patients induce osteoblastic activity in bone repair and stimulate osteocyte and bone trabecular development. Simvastatin administration is thought to reduce antioxidative inflammation, accelerate osteoinductive effect with graft, increase bone repair and significantly affect new bone formation. While graft application has a stimulating and curative effect on bone tissue and dental implant compliance, the use of simvastatin is thought to induce graft application by accelerating bone development.

## Conclusion

 It was thought that simvastatin repaired the defect area and caused a decrease in inflammation due to osteoblastic activity and increased matrix release due to increase of lamellar bone structures in rats.

## References

[1] Cassino PC, Rosseti LS, Ayala OI, Martines MAU, Portugual LC, Oliveira CG, Silva IS, Caldas RA (2018). Potencial of different hydroxyapatites as biomaterials in the bone remodeling. Acta Cir Bras.

[2] Shegarfi H, Reikeras O (2009). Review article bone transplantation and immune response. J Orthop Surg (Hong Kong).

[3] Laçin N, Kaya B, Deveci E, Kadiroglu ET, Aktas A, Yalçin M, Uysal E (2018). Comparative evaluation of ozone treatment in critical size bone defects reconstructed with alloplastic bone grafts. Int J Clin Med.

[4] Perry CR (1999). Bone repair techniques, bone graft, and bone graft substitutes. Clin Orthop Relat Res.

[5] Mundy G, Garrett R, Harris S, Chan J, Chen D, Rossini G, Boyce B, Zhao M, Gutierrez G (1999). Stimulation of bone formation in vitro and in rodents by statins. Science.

[6] Fernández-Tresguerres-Hernández-Gil I, Alobera-Gracia MA, del-Canto-Pingarrón M, Blanco-Jerez L (2006). Physiological bases of bone regeneration I Histology and physiology of bone tissue. Med Oral Patol Oral Cir Bucal.

[7] Park JB (2009). The use of simvastatin in bone regeneration. Med Oral Patol Oral Cir Bucal.

[8] Gutierrez GE, Lalka D, Garrett IR, Rossini G, Mundy GR (2006). Transdermal application of lovastatin to rats causes profound increases in bone formation and plasma concentrations. Osteoporos Int.

[9] Sakoda K, Yamamoto M, Negishi Y, Liao JK, Node K, Izumi Y (2006). Simvastatin decreases IL-6 and IL-8 production in epithelial cells. J Dent Res.

[10] Chen J, Shapiro HS, Sodek J (1992). Developmental expression of bone sialoprotein mRNA in rat mineralized connective tissues. J Bone Miner Res.

[11] Chen J, Singh K, Mukherjee BB, Sodek J (1993). Developmental expression of osteopontin (OPN) mRNA in rat tissues evidence for a role for OPN in bone formation and resorption. Matrix.

[12] Melo LG, Nagata MJ, Bosco AF, Ribeiro LL, Leite CM (2005). Bone healing in surgically created defects treated with either bioactive glass particles, a calcium sulfate barrier, or a combination of both materials A histological and histometric study in rat tibias. Clin Oral Implants Res.

[13] Ribeiro LL, Bosco AF, Nagata MJ, de Melo LG (2008). Influence of bioactive glass and/or acellular dermal matrix on bone healing of surgically created defects in rat tibiae a histological and histometric study. Int J Oral Maxillofac Implants.

[14] Alt V, Thormann U, Ray S, Zahner D, Dürselen L, Lips K, El Khassawna T, Heiss C, Riedrich A, Schlewitz G, Ignatius A, Kampschulte M, von Dewitz H, Heinemann S, Schnettler R, Langheinrich A (2013). A new metaphyseal bone defect model in osteoporotic rats to study biomaterials for the enhancement of bone healing in osteoporotic fractures. Acta Biomater.

[15] Nomura S, Wills AJ, Edwards DR, Heath JK, Hogan BL (1988). Developmental expression of 2ar (osteopontin) RNA as revealed by in situ hybridization. J Cell Biol.

[16] Oxlund H, Andreassen TT (2004). Simvastatin treatment partially prevents ovariectomy-induced bone loss while increasing cortical bone formation. Bone.

[17] Kütan E, Duygu-Çapar G, Özçakir-Tomruk C, Dilek OC, Özen F, Erdogan Ö, Özdemir I, Korachi M, Gürel A (2016). Efficacy of doxycycline release collagen membrane on surgically created and contaminated defects in rat tibiae a histopathological and microbiological study. Arch Oral Biol.

[18] Nagata MJ, Furlaneto FA, Moretti AJ, Bouquot JE, Ahn CW, Messora MR, Fucini SE, Garcia VG, Bosco AF (2010). Bone healing in critical-size defects treated with new bioactive glass/calciumsulfate a histologic and histometric study in rat calvaria. J Biomed Mater Res B Appl Biomater.

[19] Schliephake H, Zghoul N, Jäger V, van Griensven M, Zeichen J, Gelinsky M, Szubtarsky N (2009). Bone formation in trabecular bone cell seeded scaffolds used for reconstruction of the rat mandible. Int J Oral Maxillofac Surg.

[20] Truedsson A, Wang JS, Lindberg P, Gordh M, Sunzel B, Warfvinge G (2010). Bone substitute as an on-lay graft on rat tibia. Clin Oral Implants Res.

[21] Beirne OR (1986). Comparison of complications after bone removal from lateral and medial plates of the anterior ilium for mandibular augmentation. Int J Oral Maxillofac Surg.

[22] Byrd HS, Hobar PC, Shewmake K (1993). Augmentation of the craniofacial skeleton with porous hydroxyapatite granules. Plast Reconstr Surg.

[23] Corsair A (1990). A clinical evaluation of resorbable hydroxylapatite for the repair of human intra-osseous defects. J Oral Implantol.

[24] Page DG, Laskin DM (1987). Tissue response at the bone-implant interface in a hydroxylapatite augmented mandibular ridge. J Oral Maxillofac Surg.

[25] van Nieuw Amerongen GP, Vermeer MA, Nègre-Aminou P, Lankelma J, Emeis JJ, van Hinsbergh VW (2000). Simvastatin improves disturbed endothelial barrier function. Circulation.

[26] Skoglund B, Forslund C, Aspenberg P (2002). Simvastatin improves fracture healing in mice. J Bone Miner Res.

[27] Rosset EM, Bradshaw AD (2016). SPARC/osteonectin in mineralized tissue. Matrix Biol.

[28] Al-Shami R, Sorensen ES, Ek-Rylander B, Andersson G, Carson DD, Farach-Carson MC (2005). Phosphorylated osteopontin promotes migration of human choriocarcinoma cells via a p70 S6 kinase-dependent pathway. J Cell Biochem.

[29] Kariya Y, Kanno M, Matsumoto-Morita K, Konno M, Yamaguchi Y, Hashimoto Y (2014). Osteopontin O-glycosylation contributes to its phosphorylation and cell-adhesion properties. Biochem J.

[30] Duvall CL, Taylor WR, Weiss D, Wojtowicz AM, Guldberg RE (2007). Impaired angiogenesis, early callus formation, and late stage remodeling in fracture healing of osteopontin-deficient mice. J Bone Miner Res.

[31] Liao JK, Laufs U (2005). Pleiotropic effects of statins. Annu Rev Pharmacol Toxicol.

[32] Basarir K, Erdemli B, Can A, Erdemli E, Zeyrek T (2009). Osseointegration in arthroplasty can simvastatin promote bone response to implants. Int Orthop.

[33] Kheirallah M, Almeshaly H (2016). Simvastatin, dosage and delivery system for supporting bone regeneration, an update review. J Oral Maxillofac Surg Med Pathol.

[34] Nyan M, Sato D, Oda M, Machida T, Kobayashi H, Nakamura T, Kasugai S (2007). Bone formation with the combination of simvastatin and calcium sulfate in critical-sized rat calvarial defect. J Pharmacol Sci.

